# Gender Differences in Neuropsychiatric Symptoms in Mild to Moderate Alzheimer's Disease Patients Undergoing Switch of Cholinesterase Inhibitors: A *Post Hoc* Analysis of the EVOLUTION Study

**DOI:** 10.1089/jwh.2017.6420

**Published:** 2018-11-02

**Authors:** Delia Colombo, Carlo Caltagirone, Alessandro Padovani, Sandro Sorbi, Gianfranco Spalletta, Lucia Simoni, Alessandra Ori, Emanuela Zagni

**Affiliations:** ^1^ Patient Access, Novartis Farma S.p.A., Origgio, Italy.; ^2^ Neuropsychiatry Laboratory, Department of Clinical and Behavioral Neurology, IRCCS Santa Lucia Foundation, Rome, Italy.; ^3^ Department of System Medicine, University of Rome “Tor Vergata,” Rome, Italy.; ^4^ Department of Clinical and Experimental Sciences, University of Brescia, Brescia, Italy.; ^5^ Department of Neuroscience, Psychology, Drug Research and Child Health, University of Florence, Florence, Italy.; ^6^ IRCCS Don Gnocchi, Florence, Italy.; ^7^ MediNeos Observational Research, Modena, Italy.

**Keywords:** gender, Alzheimer's disease, cholinesterase inhibitors, Behavioral and Psychological Symptoms of Dementia (BPSD)

## Abstract

***Background:*** Alzheimer's disease (AD) is characterized by progressive cognitive decline, often associated with Behavioral and Psychological Symptoms of Dementia (BPSD). Acetylcholinesterase inhibitors (ChEi) may attenuate cognitive decline and mitigate BPSD. The EVOLUTION group found that the switch from oral ChEi to transdermal rivastigmine patch formulation resulted in improvement/stabilization in the frequency of clinically relevant BPSD, but gender-specific subgroup analyses were not reported.

***Methods:*** Participants underwent Neuropsychiatric Inventory to assess the frequency and severity of neuropsychiatric symptoms at baseline and 3 and 6 months after the switch from oral ChEi to transdermal rivastigmine patch. A descriptive *post hoc* analysis was conducted to assess whether there were gender-based differences in BPSD profile during the 6 months after the switch.

***Results:*** The entire sample consisted of 475 patients, 274 women and 201 men. Women were on average slightly older and with poorer cognitive performance (60.6% of the women had moderate-AD, defined as Mini-Mental State Examination [MMSE] score of 10–17, vs. 43.8% of men). In mild-AD patients (MMSE score 18–26), the frequency of BPSD did not change significantly over time and an association was found between gender and depression (odds ratio; OR [95% confidence interval; CI] female vs. male = 3.32 [1.44–7.67]), anxiety (2.42 [1.23–4.79]), apathy (2.25 [1.07–4.70]), nighttime behavior disturbances (3.97 [1.66–9.49]), and appetite/eating abnormalities (2.39 [1.10–5.18]). Moderate-AD female patients had euphoria more frequently than male patients (OR [95% CI] female vs. male = 3.67 [1.25–10.74]). The frequency of delusions, anxiety, and irritability decreased during the first 3 months after the switch, independently of gender.

***Conclusion:*** Mild-AD women tended to suffer more frequently from BPSD than men; in the 3 months after treatment switch, moderate-AD patients showed a decrease in delusions, anxiety, and irritability, with no significant differences between genders. *Ad hoc* studies to investigate this potential gender effect in AD could be well worthwhile.

## Introduction

Alzheimer's disease (AD) is an insidious illness characterized by the progressive decline of mental function; in addition to cognitive deficiencies, ∼90% of patients at any given point in the course of their illness exhibit various Behavioral and Psychological Symptoms of Dementia (BPSD).^1^ BPSD often occur in clusters: hyperactivity, including agitation and aggression; affective symptoms, including depression and anxiety; or psychosis, including both delusions and hallucinations.^2^ These symptoms are associated with poorer quality of life,^3^ a heavier burden on caregivers,^3,4^ increased risk of institutionalization,^5,6^ and increased mortality.^7^

Genders differ: women with AD suffer delusions and hallucinations more often^8,9^ and earlier^10^ than men, and depression too is more prevalent in women with AD.^9,11^

Moreover, men more commonly display apathy,^12^ aggression,^12,13^ and vegetative changes.^12^

Appropriate, tailored interventions are essential to provide quality care. Such interventions include both nonpharmacological strategies and psychotropic agents. The etiology of BPSD has not been clearly delineated, but studies assessing the benefits of acetylcholinesterase inhibitors (ChEi) on BPSD suggest that some of these symptoms, such as agitation, apathy, and psychosis, may reflect a specific central cholinergic deficiency syndrome.^14^ A meta-analysis found that 3–12 months of treatment with ChEi had a modest effect on global BPSD scores.^15^ The data on the efficacy of rivastigmine in moderating BPSD in AD patients are variable. In one 6-month study, changes in Neuropsychiatric Inventory (NPI) scores were no different between the rivastigmine and placebo groups.^16^ In a different, 12-month study, agitation/aggression and anxiety/phobias improved significantly among AD patients treated with rivastigmine.^17^

Norton et al. have suggested that caregiver interventions may become an important complement to pharmacological and other approaches to slowing the progression of dementia; they showed that for adult caregivers who are children of the patient, the personality traits of the caregivers are associated with the rate of cognitive decline in the receivers (CRs) with AD, regardless of coresidency.^18^

Furthermore, closeness and loss of closeness in the caregiver-CR dyad may be associated with either positive or adverse outcomes for caregivers.^19^

The EVOLUTION observational study evaluated the switch in ChEi in a sample of 635 AD patients who were no longer responsive to the initial treatment, with reduced tolerance and/or compliance and the loss of at least two points on their Mini-Mental State Examination (MMSE) score in the last 6 months. In analysis of a subsample of 423 patients in the EVOLUTION study, the study group found that the switch from oral ChEi to transdermal rivastigmine patch formulation not only slowed the progression of global cognitive impairment but also resulted in improvement/stabilization in the frequency of clinically relevant BPSD.^20^ While men and women are known to differ in the risk, pathogenesis, clinical manifestations, and prognosis of AD,^21–23^ the EVOLUTION study Group did not report gender-specific subgroup analyses, and current clinical guidelines do not differentiate between men and women in the use of ChEi. Accordingly, for the sample of patients in the EVOLUTION study, we conducted a *post hoc* analysis to assess whether there were gender-based differences in BPSD profile during 6 months after the switch from oral ChEi to transdermal rivastigmine patch.

## Methods

This *post hoc* analysis is part of the gender medicine project MetaGeM, designed to describe and analyze clinical outcomes, therapeutic approaches and safety data according to gender, based on nine observational studies; the MetaGeM methodology has been described extensively elsewhere.^24^

The EVOLUTION study was conducted in 38 outpatient memory clinics across Italy, on subjects who satisfied the following criteria: (1) diagnosis of probable AD according to the criteria of the National Institute of Neurological and Communicative Disorders and Stroke and the Alzheimer's Disease and Related Disorders Association (NINCDS-ADRDA)^25^; (2) mild to moderate severity of dementia, defined as MMSE^26^ score of 26 to 10; (3) onset of symptoms at least 6 months before inclusion in the sample; (4) treatment with ChEi for at least 6 months, and first switch to another type of ChEi therapy due to lack of response and/or reduced compliance (*i.e.*, lack or loss of efficacy, defined as a decline of at least two points in the MMSE score in the last 6 months) and/or reduced tolerance (due to side effects or failure to follow recommended oral dosing regimen); (5) vision and hearing sufficient for compliance with testing procedures; and (6) presence of caregiver able to understand all testing procedures. Hospitalized patients were excluded.

The EVOLUTION study design and methodology are described elsewhere.^20^ This *post hoc* analysis used a subsample of EVOLUTION patients: patients without psychiatric disorders, who switched from oral ChEi to rivastigmine transdermal patch at baseline and who underwent 3- and 6-month follow-up visits. The switch followed treatment guidelines for AD; in particular, the practice for switching called for an immediate switch from other ChEi to patch 4.6 mg/24 hours, and 1 month after the switch the dose was increased to 9.5 mg/24 hours. Patients were then kept on this dosage unless they experienced adverse events; in this case, the rivastigmine patch dosage was reduced to 4.6 mg/24 hours.

Women and men were described and compared at baseline according to sociodemographic variables (age, education, marital and occupational status, smoking habits, and alcohol consumption) and clinical features (concomitant diseases, time to onset of symptoms and to diagnosis, ChEi therapy in the 6 months before baseline). Caregivers (who remained the same for the entire length of the study) were described at baseline (age, gender, type, and cohabitation with patient) and their characteristics compared between male and female patients.

Clinical data collection included MMSE (adjusted by age and educational level for elderly patients^27^) and NPI for the assessment of BPSD, carried out at baseline and at 3- and 6-month follow-up.

The NPI is a useful instrument for characterizing the psychopathology of dementia syndromes, investigating the neurobiology of brain disorders with neuropsychiatric manifestations, distinguishing among different syndromes and assessing the efficacy of treatment.^28^ The validity and reliability of the Italian version of the NPI have been demonstrated.^29^ The NPI was used to assess the frequency and severity of neuropsychiatric symptoms in 12 domains: delusions, hallucinations, agitation, depression, anxiety, euphoria, apathy, disinhibition, irritability, aberrant motor behavior, nighttime behavior disturbances, and appetite and eating abnormalities.^24^ The severity and frequency of each symptom were scored on the basis of structured questions administered to the caregiver. Frequency was rated from 1 (only occasional) to 4 (very frequent) and severity from 1 (mild) to 3 (severe). If the symptom was absent, the score was 0. A composite score for each symptom was calculated, namely the product of the frequency and severity scores, with range accordingly from 0 to 12.

A first exploratory analysis was conducted, considering for each domain patients with available NPI scores at baseline and 3- and 6-month follow-up and comparing women and men at each study visit with regard to the presence of neuropsychiatric symptoms. BPSD were considered present for composite scores ≥1. A composite score ≥4 indicated the presence of a clinically relevant symptom typically associated with therapeutic intervention; a score of 1–3, mild symptoms usually not requiring specific treatment; and a score of 0, no symptom.^30,31^ For homogeneous gender comparison, the sample was stratified according to severity of dementia: mild-AD patients (*i.e.*, an MMSE score of 26–18) and moderate-AD patients (MMSE score of 17–10).

Comparisons by gender were performed by Student's *t*-test (for continuous variables) and *χ*^2^ or Fisher's exact test (for categorical variables). For the *post hoc* analyses, the significance threshold was set at 0.05 (all *p* values presented are exploratory, so no correction for multiple testing was applied).

Confirmatory logistic regressions for repeated measures were then run to get a further evaluation of the association between gender and BPSD. A model for each BPSD domain was estimated. As binary dependent variable, the presence (*i.e.*, a NPI composite score ≥1)/absence of each specific BPSD at time *x* (*x* = baseline, 3-month follow-up, 6-month follow-up) was considered and gender was introduced in the models as constant effect over time (no interaction between time and gender was hypothesized).

The models were then adjusted for age (≤60, 60–70, 70–80, >80 years), marital status (married/cohabiting, single, divorced/separated, widowed), education (low [none, primary], medium [junior high school], high [high school, university/postgraduate]), smoking (smoker, exsmoker, nonsmoker), alcohol consumption (none, moderate to high), concomitant ischemic heart disease, hypertension, dyslipidemia, diabetes, cerebrovascular disease (yes or no), caregiver's gender, caregiver's relationship to patient (son/daughter; husband/wife; other), ongoing antipsychotic and antidepressant therapy (yes or no), and previous treatment with oral rivastigmine (yes or no). The ongoing antipsychotic and antidepressant therapies were considered time-dependent variables and so evaluated at each time point. All the other covariates were assessed at baseline visit and considered constant over time. The results of the multivariate regressions provided an estimate of the association between gender and the presence of BPSD, adjusted for potential confounding factors.

We were also interested in estimating the effect of time on the presence of BPSD in mild- and moderate-AD patients, holding gender and all the other variables of each model constant. For this reason, the visit (baseline, 3-month follow-up, 6-month follow-up) was also included as covariate in the model.

To control for MMSE that differed significantly between genders, the models were estimated in two different subpopulations, mild- and moderate-AD patients.

Finally, we compared the results of these models with those of an exploratory analysis in which the proportion of patients with NPI remission (*i.e.*, symptoms present at baseline and absent at follow-up) was calculated and compared by gender.

Patients with missing data in some parameters were not excluded from the analysis, but only from the analysis of those parameters.

All analyses were performed with SAS v9.4 and Enterprise Guide v7.1.

## Results

### Baseline demographics, lifestyle data, and clinical characteristics

The sample analyzed consisted of a total of 475 patients: 274 women (57.7%) and 201 men (42.3%); baseline demographic data are provided in Table 1. The women were slightly older (mean ± standard deviation [SD] 77.0 ± 7.0 vs. 75.5 ± 7.0 in men, *p* = 0.022), less likely to be married/cohabiting (48.7% vs. 85.6% of men, *p*-value <0.001), and less well educated (71.2% with at most primary school vs. 61.2% of men, *p* = 0.010). Men were more likely to be past smokers (43.5% vs. 8.1% of women) or current smokers (15.5% vs. 9.6% of women, *p* < 0.001) and to be frequent alcohol consumers (38.5% vs. 15.9% of women, *p* < 0.001). No gender differences were found in employment status or concomitant diseases (except ischemic heart disease, more frequent in men [22.4%] than in women [12.4%], *p* = 0.004).

**Table 1.** Baseline Demographic, Lifestyle, and Clinical Data of MetaGem EVOLUTION Study Population

	*Men (*N* = 201)*	*Women (*N* = 274)*	p
Age, mean ± SD	75.5 ± 7.0	77.0 ± 7.0	0.022^a^
Marital status, *N* (%)^b^			
Married/cohabiting	172 (85.6)	133 (48.7)	<0.001^c^
Single	8 (4.0)	12 (4.4)	
Divorced/separated	2 (1.0)	1 (0.4)	
Widowed	19 (9.4)	127 (46.5)	
Missing		1	
Employment status, *N* (%)			
Employed	3 (1.5)	0 (0.0)	0.539^d^
Unemployed	0 (0.0)	1 (0.4)	
Retired	195 (97.0)	217 (79.2)	
Disabled	3 (1.5)	1 (0.4)	
Housewife	—	52 (19.0)	
Other	0 (0.0)	3 (1.1)	
Education, *N* (%)			
Low (none, primary school)	123 (61.2)	195 (71.2)	0.010^c^
Medium (junior high school)	32 (15.9)	45 (16.4)	
High (high school, graduate/postgraduate)	46 (22.9)	34 (12.4)	
Smoking habit, *N* (%)^b^			
Smokers	31 (15.5)	26 (9.6)	<0.001^c^
Ex-smokers	87 (43.5)	22 (8.1)	
Non-smokers	82 (41.0)	224 (82.4)	
Missing	1	2	
Alcohol consumption (from moderate to high), *N* (%)^b^	77 (38.5)	43 (15.9)	<0.001^c^
Concomitant diseases, *N* (%)	154 (76.6)	212 (77.4)	0.847^c^
Hypertension	104 (51.7)	142 (51.8)	0.986^c^
Ischemic heart disease	45 (22.4)	34 (12.4)	0.004^c^
Diabetes	28 (13.9)	42 (15.3)	0.671^c^
Dyslipidemia	32 (15.9)	46 (16.8)	0.801^c^
Cerebrovascular disease	7 (3.5)	14 (5.1)	0.394^c^
Metabolic disorders	6 (3.0)	8 (2.9)	0.967^c^
COPD	7 (3.5)	7 (2.6)	0.555^c^
Neoplasms	3 (1.5)	3 (1.1)	0.701^d^
MMSE, *N* (%)			
Mild-AD (MMSE score 18–26)	113 (56.2)	108 (39.4)	<0.001^c^
Moderate-AD (MMSE score 10–17)	88 (43.8)	166 (60.6)	

^a^ *t*-Test.

^b^ Percentages computed over non missing responses.

^c^ Chi-squared test.

For marital status, the following classes were considered: married/cohabiting versus single, divorced/separated, widowed. For alcohol consumption, the following classes were considered: no alcohol consumption, from moderate to high.

^d^ Two-sided Fisher's exact test.

For employment status, the following classes were considered: housewife/retired, other.

AD, Alzheimer's disease; COPD, chronic obstructive pulmonary disease; MMSE, Mini-Mental State Examination; SD, standard deviation.

There was no significant difference between men and women in time to onset of symptoms and to diagnosis: the means (±SD) for these variables were 1.3 (±1.1) and 2.4 (±1.7) years for men and ±1.4 (±2.2) and 2.7 (±2.0) years for women (*t*-test *p*-values, male vs. female = 0.275 and 0.159, respectively).

In the 6 months before baseline, 162, 14, and 25 men (80.6%, 7.0%, 12.4%, respectively) were treated with donepezil, galantamine, and rivastigmine (oral formulation) and 235, 24, and 15 women (85.8%, 8.8%, and 5.5%) were treated with donepezil, galantamine, and rivastigmine (oral formulation), respectively (*χ*^2^ test *p*-value, ChEi treatment male vs. female = 0.023). The daily dosage of ChEi treatment before baseline did not differ between males and females.

**FIG. 1.**
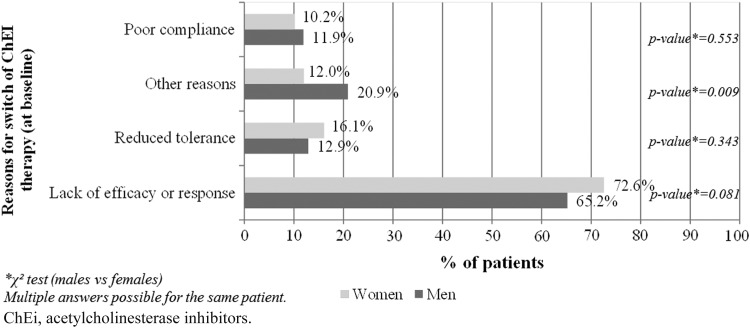
Reasons for switch of ChEi therapy at baseline by gender.

The reasons for the switch in ChEi therapy at baseline did not differ substantially between male and female patients (Fig. 1).

Among women, 5.5% were treated at baseline with memantine versus 3.6% of men (*χ*^2^ test *p*-value, male vs. female = 0.340). Women more frequently took antidepressants (sertraline, escitalopram, citalopram, venlafaxine, paroxetine) (15.3% vs. 7.5% of men; *χ*^2^ test *p*-value = 0.009), while no substantial gender differences were found at baseline in antipsychotic treatment (risperidone, olanzapine, quetiapine, ziprasidone, aripiprazole, haloperidol)—5.1% of women versus 8.5% of men; *χ*^2^ test *p* = 0.144.

**FIG. 2.**
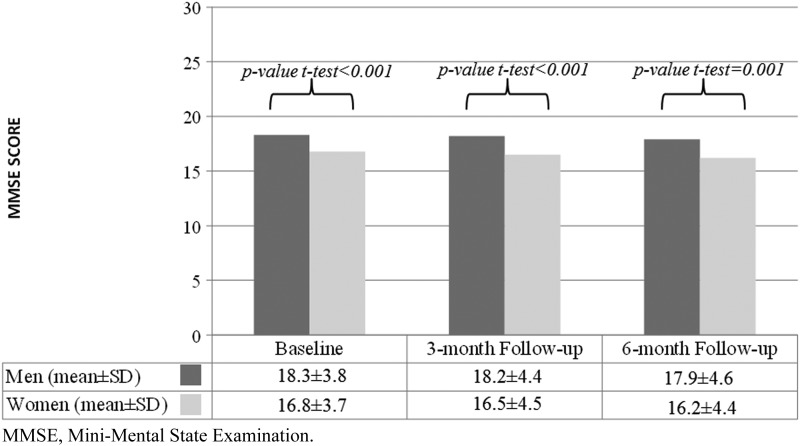
MMSE score at study visits by gender.

As shown in Table 1, 113 men (56.2%) and 108 women (39.4%) were mild-AD at baseline (MMSE score 18–26); accordingly, 43.8% of the men and 60.6% of the women were moderate-AD (MMSE score 10–17) (*χ*^2^ test *p*-value, MMSE males vs. females <0.001). At each point of measurement, women's MMSE score was significantly lower than men's (Fig. 2).

Men had a female caregiver in 89.6% of the cases versus 60.9% for female patients (*χ*^2^ test *p*-value ≤0.001); for 131 male patients (65.8%), the caregiver was the wife, while 165 female patients (61.6%) had a son or daughter as caregiver and 46 (17.2%) had other caregivers (brother/sister, daughter-/son-in-law, nephew/niece, brother-/sister-in-law, friend) (*χ*^2^ test type of caregiver, male vs. female patients, *p*-value <0.001). Other caregiver characteristics are shown in Table 2.

**Table 2.** Caregiver Characteristics at Baseline

	*Patient gender*		
	*Men (*N* = 201)*	*Women (*N* = 274)*	p
Age, mean ± SD	62.1 ± 12.9	55.8 ± 14.2	<0.001^a^
Gender, *N* (%)			
Male	21 (10.4)	107 (39.1)	<0.001^b^
Female	180 (89.6)	167 (60.9)	
Caregiver, *N* (%)^c^			<0.001^b^
Son/daughter	49 (24.6)	165 (61.6)	
Husband/wife	131 (65.8)	57 (21.3)	
Other^d^	19 (9.6)	46 (17.2)	
Missing	2	6	
Cohabiting caregiver, *N* (%)^c^			<0.001^b^
Yes	159 (79.5)	153 (55.8)	
No	41 (20.5)	121 (44.2)	
Missing	1	0	

^a^ *t*-Test.

^b^ Chi-squared test.

^c^ Percentages computed over non missing responses.

^d^ Brother/sister, daughter-/son-in-law, nephew/niece, brother-/sister-in-law, friend.

### BPSD profile in women and men

**FIG. 3.**
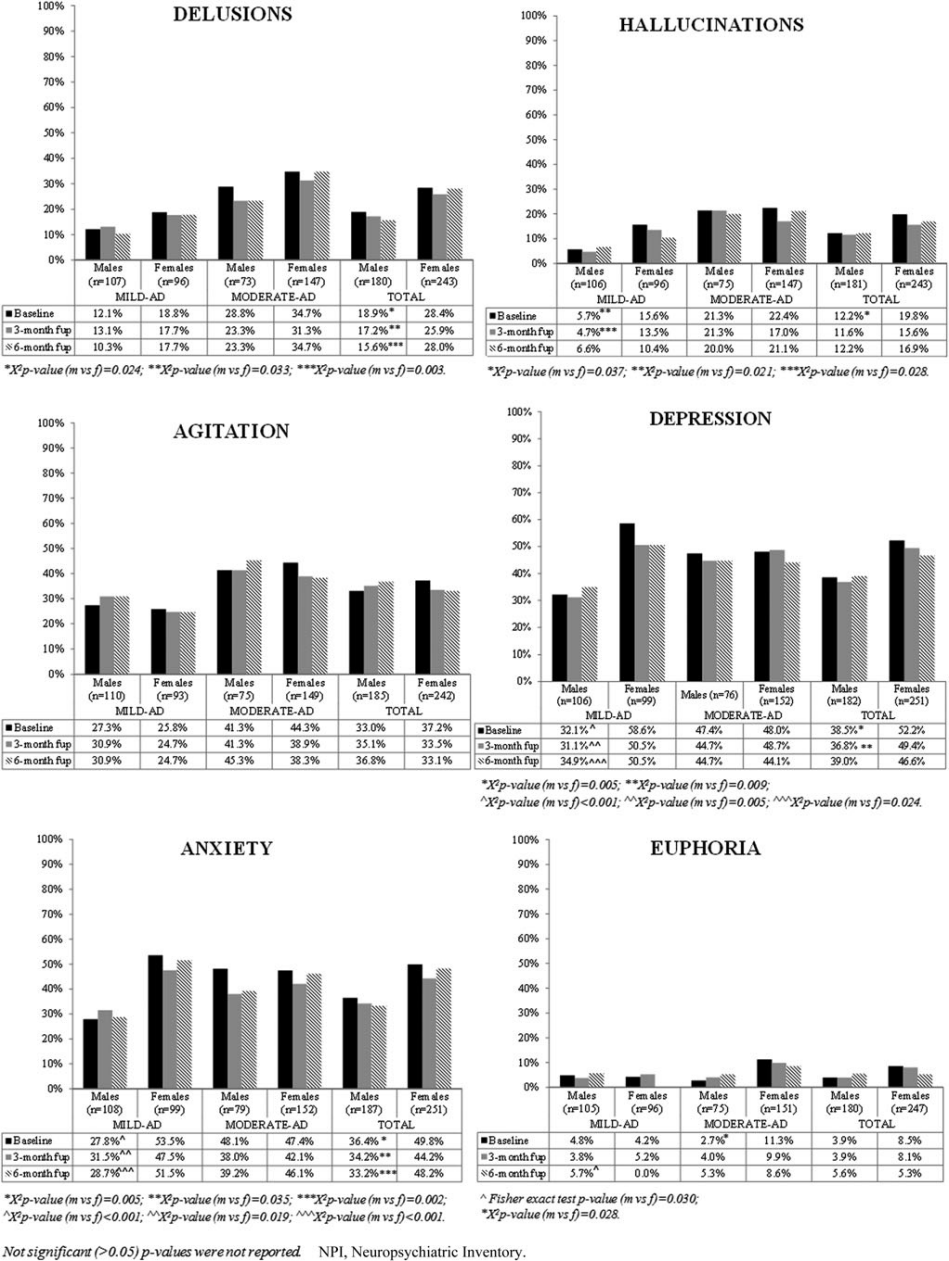
Frequency of neuropsychiatric symptoms according to NPI at study visits by gender—delusions, hallucinations, agitation, depression, anxiety and euphoria.

**FIG. 4.**
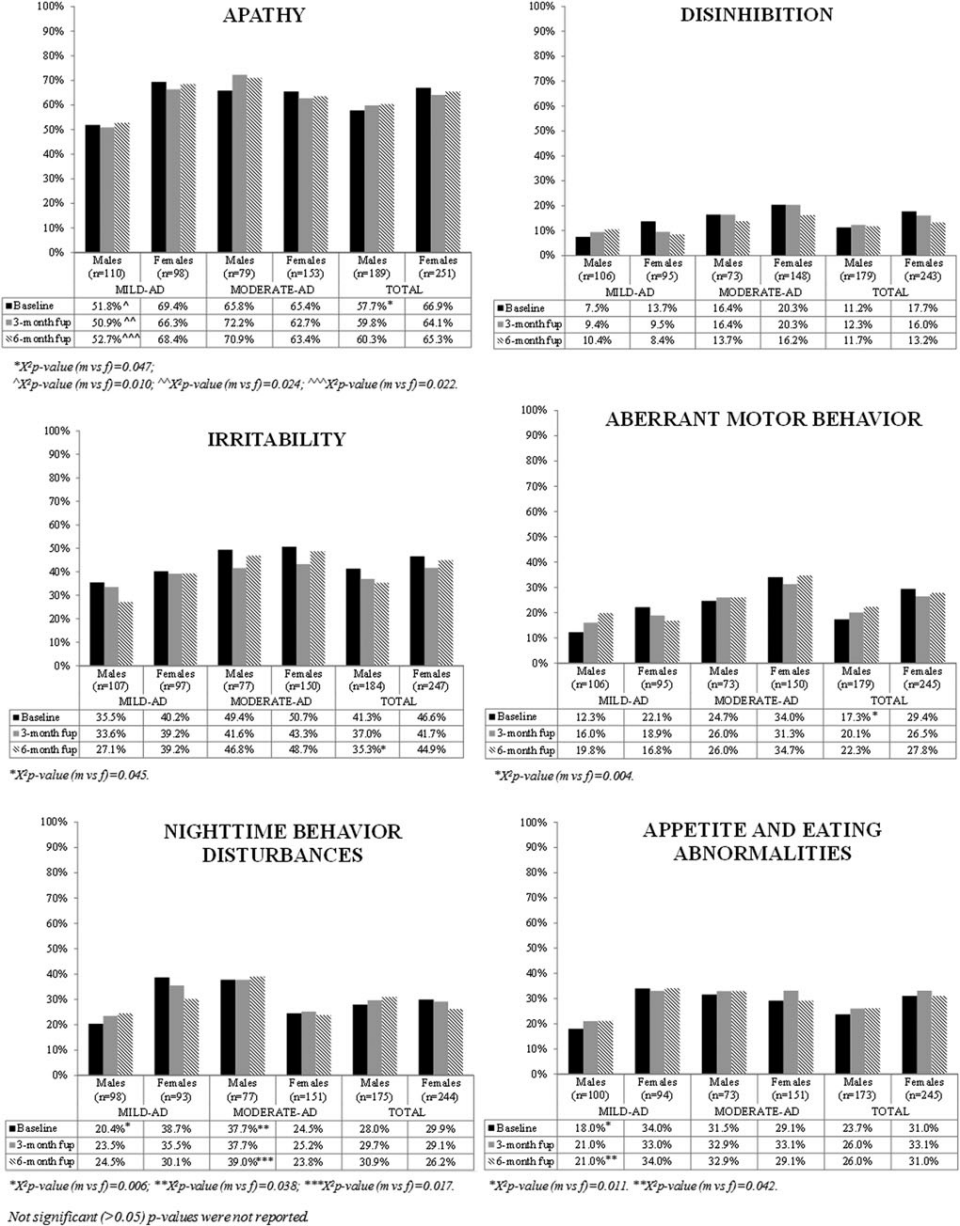
Frequency of neuropsychiatric symptoms according to NPI at study visits by gender—apathy, disinhibition, irritability, aberrant motor behavior, nighttime behavior disturbances and appetite and eating abnormalities.

Figures 3 and 4 show the results of the exploratory analysis of frequency of NPI symptoms at study visits in the entire sample and stratified by severity of symptoms (mild-AD and moderate-AD patients). The patients with missing NPI scores (not included in the analyses) did not differ significantly from those without missing scores in age, gender, or MMSE score at baseline (*p*-value *t*-test and *χ*^2^ test patients with vs. without score >0.05, data not shown).

### BPSD profile in women and men at baseline

#### Entire sample

The NPI assessment showed a higher frequency of delusions, hallucinations, depression, anxiety, apathy, and aberrant motor behavior at baseline in women than in men (see Figs. 3 and 4). No gender differences emerged in the other symptoms at baseline.

#### Breakdown between mild-AD and moderate-AD patients

For mild-AD patients, at baseline, the following symptoms were more frequent in women than men: hallucinations (15.6% vs. 5.7%, *p*-value = 0.021), depression (58.6% vs. 32.1%, *p*-value <0.001), anxiety (53.5% vs. 27.8%, *p*-value <0.001), apathy (69.4% vs. 51.8%, *p*-value = 0.010), nighttime behavior disturbances (38.7% vs. 20.4%, *p*-value = 0.006), and appetite/eating abnormalities (34.0% vs. 18.0%, *p*-value = 0.011) (see Figs. 3 and 4).

For moderate-AD patients, euphoria was more frequent at baseline in women (11.3%) than in men (2.7%, *p*-value = 0.028); differently from mild-AD patients, in moderate-AD patients nighttime behavior disturbances were more frequent at baseline in men than in women (37.7% of men vs. 24.5% of women, *p*-value = 0.038). No gender differences were observed for the other symptoms (see Figs. 3 and 4).

### BPSD profile in women and men at 3- and 6-month follow-up

#### Entire sample

The NPI assessment showed a higher frequency of delusions and depression in women than men at all points of study; anxiety too was more frequent in women than in men at 3- and at 6-month follow-up (see Fig. 3), as was irritability at 6-month follow-up (see Fig. 4).

No gender differences in the other symptoms were found at follow-up.

#### Breakdown between mild-AD and moderate-AD patients

At 3-month follow-up, mildly demented women had more frequent hallucinations, depression, anxiety, and apathy than men. At 6-month follow-up, the women suffered more than men from depression, apathy, appetite/eating abnormalities, and anxiety (see Figs. 3 and 4).

At 6-month follow-up, moderately demented men had more nighttime behavior disturbances than women (see Fig. 4).

### Association between gender and BPSD in mild- and moderate-AD patients

**FIG. 5.**
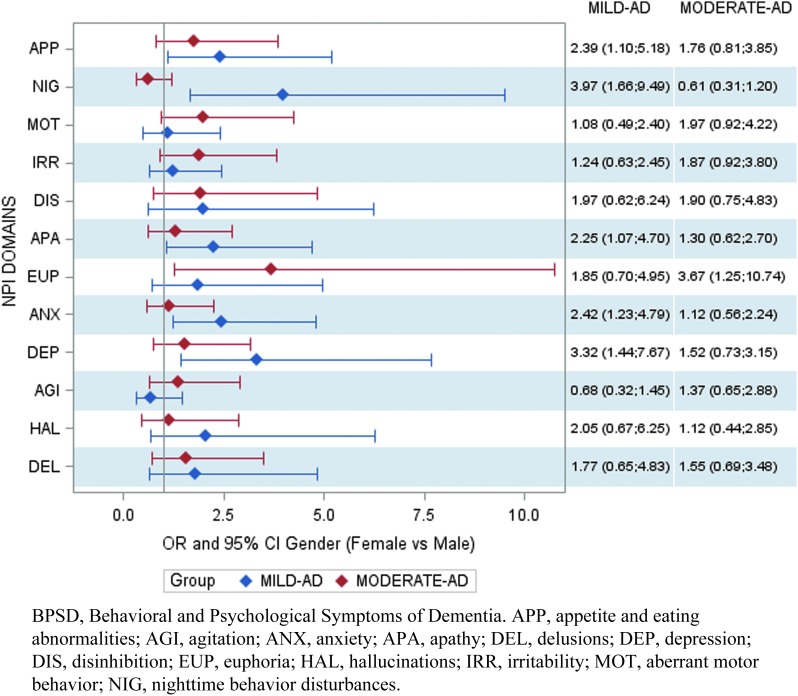
Multivariate logistic models, BPSD, and gender (odds ratios females vs. males).

In most cases, the results of the exploratory analysis were confirmed by the multivariate logistic regressions (see Fig. 5).

After controlling for all the model variables, we found an association in mild-AD patients between gender and depression (odds ratio; OR [95% confidence interval; CI] female vs. male = 3.32 [1.44–7.67]), anxiety (OR [95% CI] female vs. male = 2.42 [1.23–4.79]), apathy (OR [95% CI] female vs. male = 2.25 [1.07–4.70]), nighttime behavior disturbances (OR [95% CI] female vs. male = 3.97 [1.66–9.49]), and appetite/eating abnormalities (OR [95% CI] female vs. male = 2.39 [1.10–5.18]).

Moderate-AD female patients displayed euphoria more frequently than male patients (OR [95% CI] female vs. male = 3.67 [1.25–10.74]).

### Effect of time on BPSD in mild- and moderate-AD patients

In mild-AD patients, no significant effect of time emerged in any of the NPI domains; in this group of patients, the frequency of BPSD did not change significantly.

In moderate-AD patients, an association was found between time and delusions (OR [95% CI] at 3-month follow-up vs. baseline = 0.79 [0.64–0.98]), anxiety (OR [95% CI] visit at 3 months vs. baseline = 0.73 [0.57–0.93]), and irritability (OR [95% CI] visit at 3 months vs. baseline = 0.70 [0.53–0.93]). The frequency of delusions, anxiety, and irritability decreased during the first 3 months after the switch, independently of gender (as noted above, gender was in fact not associated with any of these domains).

No significant associations emerged between time and other BPSD.

These results (thanks to control of confounding factors through multivariate estimation) actually overcome those of our exploratory analysis about the frequency of patients with remitted NPI symptoms during follow-up (in the interests of providing fuller information, we reported results of exploratory analysis in Supplementary Table S1 (Supplementary Data are available online at www.liebertpub.com/jwh).

## Discussion

As expected,^32^ women were overrepresented, and also somewhat more cognitively impaired than men, both at baseline and throughout the 6-month follow-up period. In our sample, women and men had their therapy switched for the same reasons, proportionately, mainly lack of response to treatment.

Our *post hoc* analysis highlights more potentially relevant gender differences in BPSD. Mild-AD women were more likely than men to experience BPSD, such as depression, anxiety, apathy, nighttime behavior disturbances, and appetite/eating abnormalities, while the frequency of BPSD did not change significantly over time in the 6 months after the switch. In the moderate-AD sample, gender differences on BPSD were flattened (except for euphoria) and the frequency of delusions, anxiety, and irritability decreased during the first 3 months after the switch, independently of gender.

The female predominance in behavior disturbances was also reflected in the use of psychoactive medications, as women were more likely than men to be taking antidepressants, but not antipsychotics. Our data are in line with previous studies that found women with AD more often displaying a broader spectrum of BPSD,^33,34^ and gender was found to be a strong predictor of behavioral differences among AD patients.^12^ Women with AD were shown to suffer delusions and hallucinations more often^8,9^ and earlier^10^ than men. Furthermore, depression was associated with twice as great a risk of AD in women but not in men,^35^ and depression itself was found to be more prevalent in women with AD.^9,11^

In other studies, men have been reported as more commonly suffering apathy,^10,12^ aggression,^12,13^ and vegetative changes such as overeating and oversleeping.^12^ Other studies of AD have found a higher, but not statistically significant, incidence of aggressive behavior in men.^36,37^ Our own data confirm these findings only in part, probably owing to differences in the samples and in the tools used to assess BPSD.

Our study has a number of strengths. The sample was larger than in most previous gender studies on AD, as subjects were recruited from a number of memory clinics across Italy. Sociodemographic and clinical data were collected using uniform structured evaluations. We used a descriptive statistical analysis (with exploratory *p* values) supported by confirmatory multivariate models.

Our project assesses gender differences in BPSD profile after the switch from oral ChEi to transdermal rivastigmine patch. However, our work also has limitations. Our pooled data lack a control population. To reduce possible bias, the study considered only patients who had been switched from oral ChEi to rivastigmine patch therapy.

## Conclusions

Our study, like most previous research on AD patient populations, indicates that the prevalence of various BPSD differs between women and men. Mild-AD women tend to experience BPSD more often than men; but where cognitive dysfunction is more severe, that is, among the moderate-AD patients, the prevalence of BPSD tends to be comparable between genders. Our data also suggest that in the 3 months after the switch, moderate-AD patients experienced a decrease in delusions, anxiety, and irritability, again with no substantial gender difference. The need is for substantially increased gender-specific data on treatment response, which could carry major prognostic and therapeutic implications in clinical practice.

## Supplementary Material

Supplemental data
