# Supplementary material for: Gender Differences in Neuropsychiatric Symptoms in Mild to Moderate Alzheimer's Disease Patients Undergoing Switch of Cholinesterase Inhibitors: A Post Hoc Analysis of the EVOLUTION Study
Source: J Womens Health (Larchmt). 2018 Nov 2;27(11):1368–77. doi: 10.1089/jwh.2017.6420 (PMC6247376; doi:10.1089/jwh.2017.6420)
Supplement: Supplemental data [file Supp_Table1.pdf]

Supplementary Data

Supplementary Table S1. Frequency of Patients with Remitted Neuropsychiatric Inventory Symptoms During Follow-Up

	From baseline to	Mild-AD patients: Men, N (%)	Mild-AD patients: Women, N (%)	Mild-AD patients: p	Moderate-AD patients: Men, N (%)	Moderate-AD patients: Women, N (%)	Moderate-AD patients: p
Delusions	3-Month follow-up	5 (4.7)	8 (8.3)	0.2875[a]	5 (6.9)	10 (6.8	1.000[b]
	6-Month follow-up	6 (5.6)	9 (9.4)	0.3056[a]	7 (9.6)	12 (8.2)	0.7230[a]
	N	107 (100)	96 (100)		73 (100)	147 (100)	
Hallucinations	3-Month follow-up	3 (2.8)	5 (5.2)	0.4815[b]	**1 (1.3)**	**14 (9.5)**	**0.0215**[a]
	6-Month follow-up	**2 (1.9)**	**8 (8.3)**	**0.0496**[b]	3 (4.0)	12 (8.2)	0.2425[a]
	N	106 (100)	96 (100)		75 (100)	147 (100)	
Agitation	3-Month follow-up	**4 (3.6)**	**10 (10.8)**	**0.0462**[a]	4 (5.3)	18 (12.1)	01093[a]
	6-Month follow-up	**6 (5.5)**	**13 (14.0)**	**0.0378**[a]	5 (6.7)	19 (12.8)	0.1647[a]
	N	93 (100)	110 (100)		75 (100)	149 (100)	
Depression	3-Month follow-up	6 (5.7)	13 (13.1)	0.0653[a]	4 (5.3)	9 (5.9)	1.0000[b]
	6-Month follow-up	**5 (4.7)**	**13 (13.1)**	**0.0334**[a]	7 (9.2)	23 (15.1)	0.2125[a]
	N	106 (100)	99 (100)		76 (100)	152 (100)	
Anxiety	3-Month follow-up	8 (7.4)	11 (11.1)	0.3566[a]	10 (12.7)	20 (13.2)	0.9147[a]
	6-Month follow-up	10 (9.3)	10 (10.1)	0.8378[a]	11 (13.9)	19 (12.5)	0.7600[a]
	N	108 (100)	99 (100)		79 (100)	152 (100)	
Euphoria	3-Month follow-up	3 (2.9)	1 (1)	0.6228[b]	2 (2.7)	8 (5.3)	0.5027[b]
	6-Month follow-up	3 (2.9)	4 (4.2)	0.7114[b]	2 (2.7)	8 (5.3)	0.5027[b]
	N	105 (100)	96 (100)		75 (100)	151 (100)	
Apathy	3-Month follow-up	5 (4.6)	10 (10.2)	0.1153[a]	3 (3.8)	15 (9.8)	0.1051[a]
	6-Month follow-up	9 (8.2)	11 (11.2)	0.4575[a]	4 (5.1)	19 (12.4)	0.0757[a]
	N	110 (100)	98 (100)		79 (100)	153 (100)	
Disinhibition	3-Month follow-up	2 (1.9)	6 (6.3)	0.1524[b]	3 (4.1)	8 (5.4)	1.0000[b]
	6-Month follow-up	**2 (1.2)**	**9 (9.5)**	**0.0182**[a]	5 (6.9)	15 (10.1)	0.4233[a]
	N	106 (100)	95 (100)		73 (100)	148 (100)	
Irritability	3-Month follow-up	14 (13.1)	8 (8.3)	0.2660[a]	11 (14.3)	21 (14.0)	0.9533[a]
	6-Month follow-up	15 (14.0)	13 (13.4)	0.8983[a]	12 (15.6)	22 (14.7)	0.8544[a]
	N	107 (100)	97 (100)		77 (100)	150 (100)	
Aberrant motor behavior	3-Month follow-up	**1 (0.9)**	**11 (11.6)**	**0.0015**[a]	5 (6.9)	15 (10.0)	0.4397[a]
	6-Month follow-up	**1 (0.9)**	**12 (12.6)**	**0.0008**[a]	7 (9.6)	18 (12.0)	0.5923[a]
	N	106 (100)	95 (100)		73 (100)	150 (100)	
Nighttime behavior disturbances	3-Month follow-up	3 (3.1)	9 (9.7)	0.0596[a]	4 (5.2)	16 (10.6)	0.1727[a]
	6-Month follow-up	**6 (6.1)**	**14 (15.1)**	**0.0439**[a]	7 (9.1)	19 (12.6)	0.4327[a]
	N	98 (100)	93 (100)		77 (100)	151 (100)	
Appetite and eating abnormalities	3-Month follow-up	6 (6.0)	11 (11.7)	0.1604[a]	4 (5.5)	10 (6.6)	1.0000[b]
	6-Month follow-up	6 (6.0)	10 (10.6)	0.2405[a]	3 (4.1)	17 (11.3)	0.0786[a]
	N	100 (100)	94 (100)		73 (100)	151 (100)	

Results with p-values < 0.05 are in bold.

Percentages computed within class (mild-AD men, mild-AD women, moderate-AD men, moderate-AD women) over the total number of patients with available score within each domain.

[a]χ^2 test (males vs. females).

[b]Two-sided Fisher's exact test (males vs. females).

AD, Alzheimer's disease; NPI, Neuropsychiatric Inventory.
